# Porcine DNAJB6 promotes PCV2 replication via enhancing the formation of autophagy in host cells

**DOI:** 10.1186/s13567-020-00783-z

**Published:** 2020-05-07

**Authors:** Cong Han, Qian Du, Lei Zhu, Nannan Chen, Le Luo, Qiao Chen, Jiatong Yin, Xingchen Wu, Dewen Tong, Yong Huang

**Affiliations:** College of Veterinary Medicine, Northwest A&F University, Yangling, China

## Abstract

Hsp40/DnaJ family proteins play important roles in the infection process of various viruses. Porcine DNAJB6 (pDNAJB6) is a major member of this family, but its role in modulating the replication of porcine circovirus type 2 (PCV2) is still unclear. In the present study, pDNAJB6 was found to be significantly upregulated by PCV2 infection, and confirmed to be interacted with PCV2 capsid (Cap) protein and co-localized at both cytoplasm and nucleus in the PCV2-infected cells. Knockout of pDNAJB6 significantly reduced the formation of autophagosomes in PCV2-infected cells or in the cells expressing Cap protein, whereas overexpression of pDNAJB6 showed an opposite effect. In addition, the domain mapping assay showed that the J domain of pDNAJB6 (amino acids (aa) 1–99) and the C terminus of Cap (162-234 aa) were required for the interaction of pDNAJB6 with Cap. Notably, the interaction of pDNAJB6 with Cap was very important to promoting the formation of autophagosomes induced by PCV2 infection or Cap expression and enhancing the replication of PCV2. Taken together, the results presented here show a novel function of pDNAJB6 in regulation of porcine circovirus replication that pDNAJB6 enhances the formation of autophagy to promote viral replication through interacting with viral capsid protein during PCV2 infection.

## Introduction

Porcine circovirus associated diseases (PCVAD), caused by the porcine circovirus type 2 (PCV2), is one of the widespread infection diseases in the global swine industry. PCV2 belongs to the genus *Circovirus* within the family Circoviridae. The genome of PCV2 composed of 60-copies of capsid protein is a single-stranded, no-segmented and closed-circular DNA with 1.7 kb in size and 20 nm in diameter [1, 2]. The virus genome contains 11 open reading frames (ORFs) [3], and ORF1 encodes replication associated proteins (Rep, Rep’, Rep3a, Rep3b, Rep3c, NS515, NS672 and NS’) for the rolling circle replication of the genome [4]. ORF2 encodes another major structural protein, which is the unique capsid protein (Cap) and the important epitope of PCV2 [5]. ORF3 encodes the apoptotic associated protein, and ORF4 encodes antiapoptotic protein (ORFs) [3, 6, 7].

As the member of the Circoviridae, Cap plays multiple roles in the replication of PCV2. The N-terminal part of Cap displays a nuclear localization signal (NLS), which is required for the proper localization of Cap during the viral cycle. It has been shown that Cap transported into the nucleus by NLS for enclosing the genome and forming the capsid to assemble infectious virion [8–10]. Virion then egressed from the infective cell to initiate another cycle of infection [2]. In addition, PCV2 Cap was shown to interact with the viral replicases, which are required for virus replication [11]. In recent years, many reports have demonstrated that numerous host proteins interact with PCV2 Cap, as well as being part and parcel during the infection and replication of the virus. For example, PCV2 Cap interacts with porcine Makorin RING finger protein (MKRN1), and MKRN1 modulates the replication and pathogenesis of PCV2 through E3 ligase-medicated ubiquitination and degradation of Cap [5]. Moreover, C1q receptor (gC1qR), Heat shock protein 40 (Hsp40), Nucleophosmin 1 (NPM1), prostate apoptosis response-4 (Par-4) and nucleosome assembly protein 1(Nap1) are all interacting proteins of PCV2 Cap [5, 12]. Until now, most of the studies have been focusing on the roles of PCV2 Cap protein in viral genomic replication or virulence, but the specific roles of host proteins interacting with Cap during PCV2 replication are still rare.

Hsp40 or DnaJ is an evolutionarily conserved family of proteins involved in folding and translocation of cellular proteins, assembly of multi-protein complex, degradation of misfolded proteins, and regulation of various viral replications [13–15]. According to their domain structures, DnaJ can be classified into 3 groups (A, B, and C) [16]. Notably, some of these subtypes promote the replication of different viruses, while others play suppressive roles. Human Hdj2, a member of the Hsp40 (A1) subfamily, facilitates replication of Japanese encephalitis virus (JEV) by interacting with JEV nonstructural protein 5 (NS5) [17]; Human Hsp40B1 promotes herpes simplex virus type 1 (HSV-1) replication by enhancing the binding of HSV-1 origin-binding protein (UL9) to the origin of viral DNA replication [18]; Hsp40 activation is critical to adenovirus (AdV) replication [19]; Hsp40B6/Hdj1 facilitates the nuclear import of the human immunodeficiency virus type 2 (HIV-2) Vpx-mediated preintegration complex [20]; Hsp40B1 promotes influenza A virus replication by assisting nuclear import of viral ribonucleoproteins [21]; Simian virus 40 (SV40) infection required Hsp40B11, B12, B14, and C18, while BiP formed a complex with SV40 capsids in the ER in a DNAJB11-dependent fashion [22]. These studies identified Hsp40 as a positive regulator of viral replication. By contrast, it has been reported that Hsp40A1, B1, B6, and C5 instead of C3 can limit HIV-1 production [13]. Hsp40A3/Hdj2 and Hsp40B6/Hdj1 negatively regulate hepatitis B virus replication [15]. However, the role of Hsp40 in the PCV2 life cycle remains to be studied.

Autophagy plays an important role in the occurrence and development of some diseases [23, 24]. On the one hand, autophagy acts as a host defense mechanism against viruses through degradation of the components of viruses or regulation of either innate or adaptive immune responses [25, 26]. For instance, the important structural protein of foot-and-mouth disease virus VP1 is degraded by the autophagolysosomal pathway [27]. On the other hand, some viruses use host autophagy for replication [28–31]. Previous reports indicate that PCV2 and its capsid protein can promote the formation of autophagosome and autophagic flux in PK-15 cells and further enhance their replication [23]. Based on previous reports, we aimed at further identifying the roles of host protein pDNAJB6 in the formation of autophagy induced by PCV2.

In this study, we found that knockout of Hsp40/pDNAJB6 significantly reduced the production of progeny virion in PCV2 infected cells, and significantly reduced the formation of autophagosomes induced by either PCV2 infection or Cap protein. Overexpression of Hsp40/pDNAJB6 markedly increased the number of autophagosomes and further promoted production of progeny virion in PCV2-infected cells, whereas inhibition of autophagic formation blocked the enhancement effect of Hsp40/pDNAJB6 on PCV2 replication. Furthermore, we found that in the Hsp40/pDNAJB6 deficient cells, expression of mutated Hsp40/pDNAJB6, deleted in the critical binding domain interacting with Cap, could not increase the formation of autophagy and progeny virion production. These findings demonstrated that pDNAJB6, as Cap-binding protein, influenced PCV2 replication by promoting autophagy formation.

## Materials and methods

### Cells, virus and reagents

PK-15 (Porcine kidney 15 cell lines) were donated from the Innovative team of animal pathogen surveillance and epidemiology in Harbin Veterinary Research Institute (CAAS, Heilongjiang, China); HEK 293T (human embryonic kidney 293 cells transfected with SV40 large T-antigen) cells were purchased from the American Type Culture Collection (ATCC, Manassas, VA, USA). These cells were cultured in Dulbecco modified Eagle medium (DMEM; Invitrogen, Carlsbad, CA, USA). All media were supplemented with 10% heat-inactivated fetal bovine serum (FBS; Sijiqing, Hangzhou, China), and maintained at 37 °C with 5% CO_2_. The PCV2 strain (GenBank No. MH492006) was isolated and stocked in our lab.

Rabbit monoclonal anti-DNAJB6 antibody was purchased from (ab198995; abcam, Cambridge, MA, UK). Anti-PCV2 Cap antibody was produced in rabbit by our team. Rabbit monoclonal anti-GFP tag antibody (G10362) and rabbit monoclonal anti-Flag tag (701629) antibody were purchased from Thermo Fisher (Waltham, MA, USA). Mouse monoclonal antibody against GFP tag was purchased from CUSABIO (CSB-MA000051M0m; Wuhan, China). Mouse monoclonal anti-Flag (M2) antibody was obtained from Sigma-Aldrich (F1804; St. Louis, MO, USA). Rabbit monoclonal anti-His tag antibody was purchased from Cell Signaling Technology (D3I10; Danvers, MA, USA).

Anti-GST tag mouse monoclonal antibody was purchased from ABways (AB0003; Shanghai, China). β-actin mouse monoclonal antibody was purchased from GenScript (A00702; Nanjing, China). HRP-conjugated goat anti-mouse IgG (RK244131) and anti-rabbit IgG (RJ242536) were produced at Invitrogen.

Protein G-agarose and protein A-agarose were purchased from Santa Cruz; 3-MA (M9281) was purchased from Sigma-Aldrich. For the analysis of autophagosome, PK-15 or PK-15/EGFP-LC3 cells were pretreated with 5 mM of 3-methyladenine (3-MA, Cat: M9281, Sigma-Aldrich) for 2 h at 37 °C. The cells were then infected with the indicated titer of PCV2, and further incubated in fresh media in the absence or presence of 3-MA (5 mM), or the corresponding amount of the solvent dimethyl sulfoxide (DMSO, D8370; Solarbio Life Sciences, Beijing, China).

### Plasmids construct and siRNA

PCV2 Cap encoding gene and three truncated mutant fragments were amplified from PCV2 genomes template and subcloned into the pEGFP-N1 or pCI-neo vector. The encoding gene of porcine DNAJB6 was amplified from the cDNA of PK-15 cells and subcloned into the pCI-neo vector with or without a Flag tag sequence. Meanwhile, full-length porcine DNAJB6 and three truncated DNAJB6 fragments were subcloned into the pGEX-4T-1. Recombinant plasmid pET-28a-Cap was stored in our lab [32]. The PCR primers used in this study were shown in Table 1. All sequences in constructs were confirmed by sequencing analysis (Sangon Biotech, Shanghai, China).

**Table 1:** **Primers used in this study**

Primers	Sequences (5′–3′)	Vectors
DNAJB6-F	CGGAATTCATGGTAGATTACTATGAAG	pGEX-4T-1
DNAJB6-R	CCGCTCGAGCTAGGGGCGGCCCTTGGTCGA	
DNAJB6_1-99_-F	CGGAATTCATGGTAGATTACTATGAAG	pGEX-4T-1
DNAJB6_1-99_-R	GCGTCGACCTATTCCCTGGAGACGTCATC	
DNAJB6_73-181_-F	CGGAATTCGCCACCATGGGATTGAACGGTGGCAGTG	pGEX-4T-1
DNAJB6_73-181_-R	GCGTCGACCTAACCAAACGCCGTGGAAGAGAAG	
DNAJB6_100-327_-F	CGGAATTCGCCACCATGAGGGAATTTTTTGGTGGAAG	pGEX-4T-1
DNAJB6_100-327_-R	CCGCTCGAGCTAGGGGCGGCCCTTGGTCGA	
F-DNAJB6-F	CGGAATTCATGGATTACAAGGATGACGACGATAAGGGAGG CATGGTAGATTACTATGAAG	pCI-neo
F-DNAJB6-R	GCTCTAGACTAGGGGCGGCCCTTGGTCGA	
P-DNAJB6_1-99_-F	CGGAATTCATGGATTACAAGGATGACGACGATAAGGGA GGCATGGTAGATTACTATGAAG	pCI-neo
P-DNAJB6_1-99_-R	GCTCTAGACTATTCCCTGGAGACGTCATC	
P-DNAJB6_73-181_-F	CGGAATTCATGGATTACAAGGATGACGACGATAAGG	pCI-neo
P-DNAJB6_73-181_-R	GCTCTAGACTAACCAAACGCCGTGGAAGAGAAG	
P-DNAJB6_100-327_-F	CGGAATTCATGGATTACAAGGATGACGACGATAAGGGAGGCATGAGGGAATTTTTTGATGG	pCI-neo
P-DNAJB6_100-327_-R	GCTCTAGACTAGGGGCGGCCCTTGGTCGA	
Cap-F	CCGCTCGAGATGACGTATCCAAGGAGGCGT	pEGFP-N1
Cap-R	CCCAAGCTTAGGGTTAAGTGGGGGGTC	
Cap_1-161_-F	CCGCTCGAGATGACGTATCCAAGGAGGCGT	pEGFP-N1
Cap_1-161_-R	CCCAAGCTTAAGTACCGGGTGTGGTAGGAG	
Cap_118-234_-F	CCGCTCGAGGTTGGATCCAGTGCTGTTATTC	pEGFP-N1
Cap_118-234_-R	CCCAAGCTTAGGGTTAAGTGGGGGGTC	
Cap_162-234_-F	CCGCTCGAGCCCAAACCTGTCCTTGATTCC	pEGFP-N1
Cap_162-234_-R	CCCAAGCTTAGGGTTAAGTGGGGGGTC	
Cap-F	CCGCTCGAGATGACGTATCCAAGGAGGCGT	pCI-neo
Cap-R	CCCGTCGACAGGGTTAAGTGGGGGGTC	

Restriction enzyme sequences are underlined.

### siRNA and transfection

Specific siRNAs were designed to silence *atg5*. The sequences of *atg5* (atg5#1: GGAUGUAAUUGAAGCUCAUTT, atg5 #2: AUGAGCUUCAAUUACAUCCTT) were referred to previous article published in 2012 [23]. The effects of siRNA were identified by western blot. Negative control siRNA or specific siRNA were transfected into target cells using the Lipo6000 Transfection Reagent (C0526; Beyotime, Shanghai, China). The cells were grown at 37 °C for 24 h and subsequently infected with PCV2 for 48 h. In addition, all the eukaryotic expression plasmids were transfected into target cells also using the Lipo6000 Transfection Reagent (C0526; Beyotime).

### CRISPR/cas9 KO cell

Targeting sites in the *pDNAJB6* gene were selected using the CRISPR program (Genome Engineering. Broad Institute Cambridge, MA, USA). Oligonucleotides for the target sequences were annealed and cloned into the *Bsm*B I sites of lenti-CRISPRv2 plasmid (Addgene, #52961), and were transfected into HEK293T cells together with the packaging plasmids psPAX2 (Addgene, #12260) and pMD2.G (Addgene, #12259) to generate the recombinant lentivirus (Re-Lenti232, Re-Lenti845). 72 h post-transfection, cell culture supernatants were harvested by 13 000 *g* centrifugation, and the titers of the lentivirus were determined by qPCR [5]. PK-15 cells were infected with the recombinant lentivirus (Re-Lenti232, Re-Lenti845) for 48 h, and selected with puromycin (5 μg/mL) for about two weeks. Then, positive cells were obtained and subcloned into 96-well plates for single-clone cells.

### Construction of pDNAJB6 overexpression cell lines

PK-15 cells were transfected with pCI-Flag-DNAJB6 or pCI-neo using the Lipo6000 Transfection Reagent (Beyotime). The medium was replaced by Dulbecco modified Eagle medium (DMEM, Invitrogen, USA) with 10% heat-inactivated fetal bovine serum (Sijiqing, China) and 0.5 mg/mL G418 (Solarbio Life Sciences, G8161) after 5 h for selected PK-15^DNAJB6^ and PK-15^PCI^ cells. The overexpression of pDNAJB6 was determined by Western blotting using anti-Flag antibodies or anti- DNAJB6 antibodies.

### Real-time PCR

According to Invitrogen manufacturer, total cellular RNA was extracted with TRIzol. The cDNA was synthesized from the RNA samples using HiScript II Q RT SuperMix for qPCR (R223-01; Vazyme, Nanjing, China). PCV2 DNA was extracted by MiniBEST Viral DNA Extraction Kit (BioTeke, Beijing, China) according to the manufacturer’s instructions. Quantitative PCR analysis was performed on the iQ5 real-time PCR System (Bio-Rad, Hercules, CA, USA) using ChamQ Universal SYBR qRCR Master Mix (Q711-02; Vazyme). The pDNAJB6 mRNA transcription levels was normalized against that of porcine β-actin by 2-ΔΔCt method, the relative fold change of pDNAJB6 mRNA at different time point of PCV2 infected or mock-infected PK-15 cells were then calculated. All test samples were run in three independent experiments. Data are representative of the mean values. PCV2 qPCR and β-actin primer sequences were used as previous description [5]. DNAJB6 qPCR primer sequences were: DNAJB6-F: ATGAGGGAATTTTTTGATGGAAGGG; DNAJB6-R: CTAATTTTCCTGCCGTTGACCATTTTAG.

### Co-immunoprecipitation (co-IP) Assays

HEK-293T cells were cultured in 10 cm cell plate, and the cells were transfected with the indicated plasmids using Lipo6000 Transfection Reagent. After 36 h, the cells were lysed with lysis Buffer (150 mM NaCl, 50 mM Tris–HCl [pH 7.4], 1% Nonidet P-40, 0.5% TritonX-100, 1 mM EDTA, 0.1% sodium deoxycholate, 1 mM dithiothreitol, 0.2 mM phenylmethylsulfonyl fluoride, and a protease inhibitor protease inhibitor cocktail [Sigma-Aldrich]) on ice for 40 min. Next, it was centrifuged at 13 000 *g* 15 min at 4 °C to obtain the lysate supernatant. The lysate supernatant was incubated with G-agarose/protein A-agarose (Santa Cruz, CA, USA) for 1.5 h at 4 °C. After centrifugation at 13 000 *g*, 10 min at 4 °C, the supernatant was incubated with the indicated antibody at 4 °C overnight. Then, the corresponding G-agarose/protein A-agarose was added to the above mixture and incubated for 6 h at 4 °C, before centrifugation at 15 000 *g* for 1 min to obtain a precipitate and 3 washes with PBS. Finally, the proteins were eluted by boiling for 5 min in 5 × loading buffer and analyzed by Western blot.

### GST Pull-Down assay

HEK293T cells were transfected with pEGFP-Cap or each of its truncated mutants using Lipo6000 transfection reagent and harvested 48 h post-transfection. Cells were lysed with lysis Buffer (150 mM NaCl, 50 mM Tris–HCl [pH 7.4], 1% Nonidet P-40, 0.5% TritonX-100, 1 mM EDTA, 0.1% sodium deoxycholate, 1 mM dithiothreitol, 0.2 mM phenylmethylsulfonyl fluoride, and a protease inhibitor protease inhibitor cocktail [Sigma-Aldrich]) on ice for 40 min. After centrifugation for 15 min 12 000 *g* at 4 °C, the lysate supernatants were incubated with purified GST protein for 3 h at 4 °C. Glutathione Beads 4FF (SA010010; Smart-Life Sciences, Changzhou, China) which has been pre-washed 3 times with PBS, was then added, incubated for 1 h at 4 °C, before centrifugation at 1500 *g* for 3 min to obtain the supernatant for subsequent using. In addition, after the prokaryotic expression of pET-28a-Cap, the His tagged Cap protein was purified for subsequent using.

For GST-pull down assays, GST, GST-DNAJB6 or GST-DNAJB6 deletion mutants protein produced in *E. coli* (BL21) cells were incubated with Glutathione Beads, which was pre-washed 3 times with PBS, at 4 °C overnight. After 3 washes with buffer A (20 mM Tris–HCl [pH 8.0], 150 mM NaCl, 1 mM MgCl_2_, 0.1% Nonidet P-40, 10% glycerol, 0.1 mM dithiothreitol, and a protease inhibitor), all of the beads were incubated with the indicated proteins for 3 h at 4 °C, and followed by washing 3 times with the buffer A. Finally, the proteins were eluted by boiling for 10 min in 5 × loading buffer and detected by immunoblotting.

### Confocal microscopy

PK-15 cells were grown on coverslips in 24-well culture plates and transfected with the indicated plasmids. All the cells were rinsed with pre-cooled 0.1 M PBS three times and fixed with 4% paraformaldehyde for 60 min at room temperature. Next, cells were permeabilized with 0.1% Triton X-100 for 10 min at 37 °C and then washed 3 times with PBS. After blocking with 5% BSA, the samples were incubated with the indicated primary antibodies overnight at 4 °C and with the secondary antibody (Dylight 594 goat anti-rabbit IgG (H+L) antibody, Genshare biological, Xi’an, China, JC-PB007D) for 2 h at 37 °C, followed by washing 3 times with PBS. Then, the cells were stained with DAPI (Beyotime) at 37 °C for 15 min and washed as described above. Finally, coverslips were mounted on glass slides, and the image was captured by a laser scanning confocal microscope (LECIA TCS SP8, Germany).

In addition, PK-15 cells were transiently transfected with EGFP-LC3 using Lipo6000 Transfection Reagent; the medium was replaced by DMEM with 10% FBS after 6 h, and the cells were incubated for 12 h. Then, cells were transfected with pCI-Cap or infected with PCV2 for the indicated time. Cells were fixed and permeabilized as described above. Then, the samples were stained with DAPI at 37 °C for 15 min and washed 3 times with PBS. Cells were analyzed by laser scanning confocal microscope, and images were recorded by Leica X version software. The average number of EGFP-LC3 puncta per cell was counted from at least 60 cells in per sample [23].

## Results

### PCV2 infection upregulates pDNAJB6 expression in host cells

**Figure 1:**
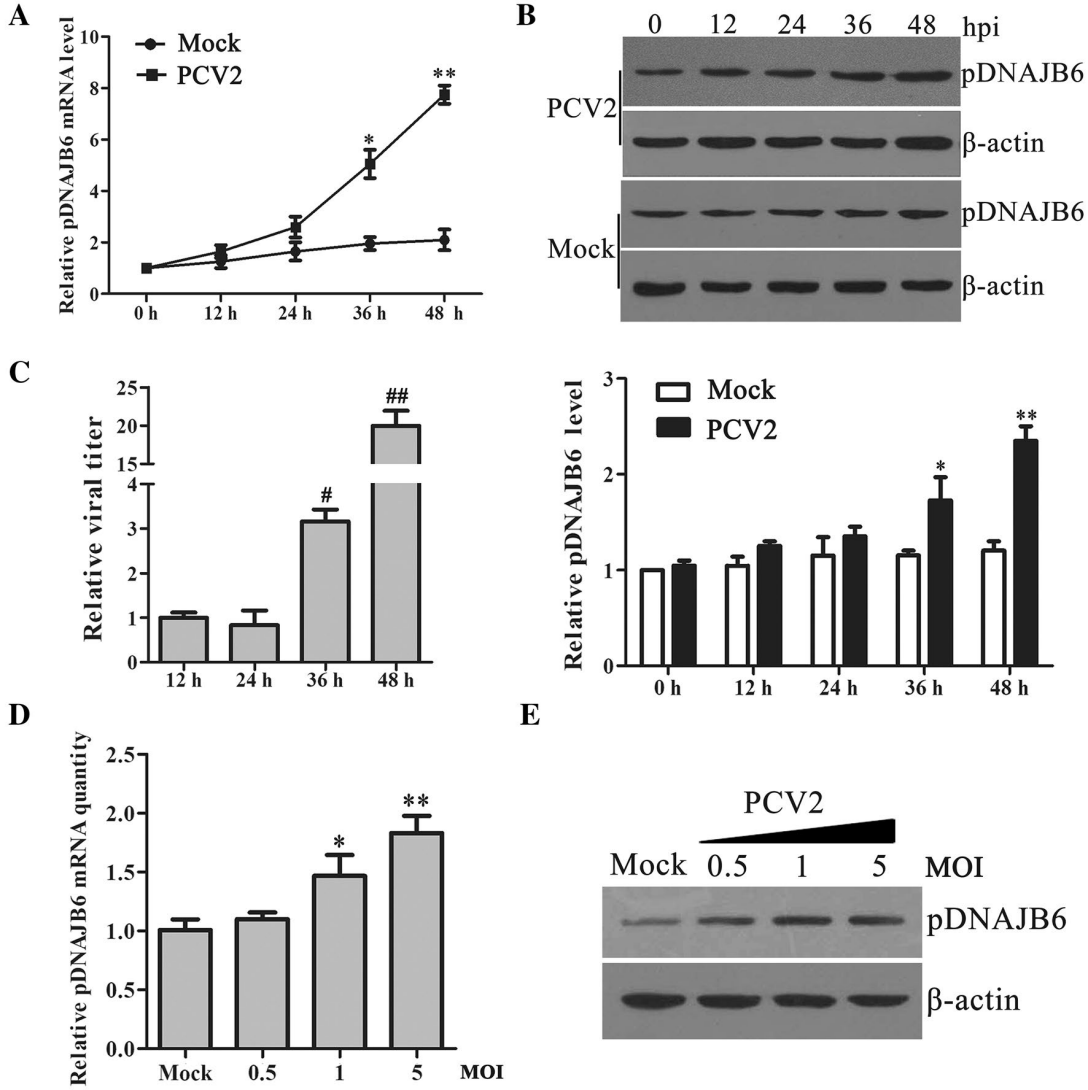
**PCV2 infection upregulates pDNAJB6 expression in PK-15 cells. A–C** PK-15 cells were infected with 1 MOI of PCV2 or mock infection for indicated time, the mRNA level of pDNAJB6 (**A**), the protein level of pDNAJB6 (**B**) and the relative viral titers of different infected time were measured by TCID_50_ (**C**). **D, E** qPCR and Western blot analysis of pDNAJB6 mRNA and protein levels in PK-15 cells infected with different doses of PCV2 (MOI = 0, 0.5, 1, or 5) for 36 h. β-actin served as an internal control. Results are shown as the mean ± SD (n = 3). *p < 0.05, **p < 0.01 versus mock infected cells at same time points; ^#^*p* < 0.05, ^##^*p* < 0.01 versus cells infected with PCV2 for 24 h.

Hsp40/DnaJ, an evolutionarily conserved family of proteins, has been identified to be involved in the regulation of various viral replication [13, 16, 17, 21]. Porcine DNAJB6 (pDNAJB6), as an important member of Hsp40/DnaJ family, also has been identified to be involved in the infectious process of some porcine viruses [27]. To determine the roles of pDNAJB6 in PCV2 replication, we firstly investigated the expression profiles of pDNAJB6 in PCV2-infected PK-15 cells. As shown in Figures 1A–C, in the cells infected with 1 MOI of PCV2, pDNAJB6 mRNA began to markedly increase at 36 h post-infection (hpi) compared with mock-infected cells, and further increased at 48 hpi; pDNAJB6 protein also showed an increased tendency at 24 hpi, and indeed appeared a significant increase from 36 hpi relative to mock-infected cells. In line with the changes of pDNAJB6 in infected cells, PCV2 copies further markedly increased at 36 hpi relative to 24 hpi (Figure 1C). To further confirm the effects of PCV2 infection on the expression of pDNAJB6, PK-15 cells were infected with different doses of PCV2 (MOI = 0, 0.5, 1, or 5) for 36 h. The results showed that pDNAJB6 expression significantly increased with the doses of PCV2 infection, suggesting that the expression level of pDNAJB6 is associated with viral loads (Figures 1D and E). These results demonstrated that the expression of pDNAJB6 is upregulated by PCV2 infection.

### PCV2 Cap interacts with pDNAJB6 and co-localizes in both cytoplasm and nucleus

**Figure 2:**
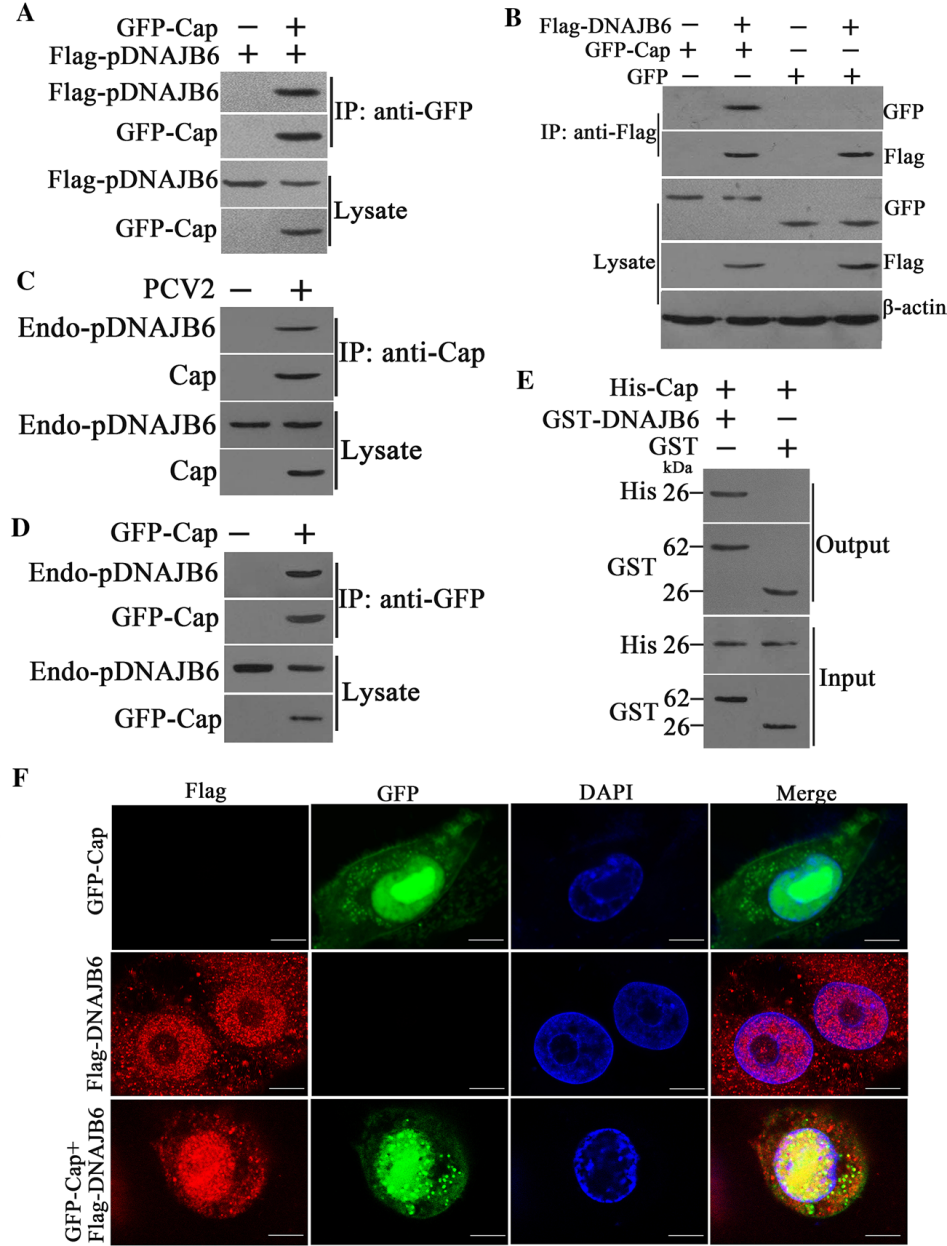
**PCV2 Cap interacts with pDNAJB6 and co-localizes in both cytoplasm and nucleus. A, B** PCV2 Cap interacts with pDNAJB6 in transfected cells. HEK293T cells were co-transfected with GFP-Cap and Flag-pDNAJB6 expression plasmids or co-transfected with the pEGFP-N1 control vector and the Flag-pDNAJB6 expression plasmid. The cells transfected with pEGFP-N1 control vector, GFP-Cap expression plasmid or Flag-pDNAJB6 expression plasmid alone served as controls. The interaction of GFP-Cap with Flag-pDNAJB6 was identified through immunoprecipitation using anti-Flag antibodies or anti-GFP antibodies. **C** pDNAJB6 interacts with PCV2 Cap protein. PK-15 cells were infected with PCV2 (MOI = 1) for 36 h, and cell lysates were immunoprecipitated using Cap antibodies followed by immunoblots using pDNAJB6 antibodies. **D** Endogenous pDNAJB6 interacts with GFP-Cap. PK-15 cells were transfected with GFP-Cap, and the cells were lysed for immunoprecipitation with GFP antibodies; pDNAJB6 antibodies were used for immunoblotting. In these co-IP assay, the corresponding whole cell lysate (Lysate) were detected the targeted protein before immunoprecipitation. **E** Direct interaction of pDNAJB6 with PCV2 Cap. Bacterially purified GST-pDNAJB6 or GST alone was incubated with purified His-Cap, and these input proteins (Input) and the proteins pulled-down by glutathione Sepharose beads (Output) were analyzed by Western blot with the indicated antibodies. **F** pDNAJB6 protein co-localizes with Cap. PK-15 cells were co-transfected with GFP-Cap and Flag-pDNAJB6 expression plasmid, and transfected with pEGFP-N1 and pCI-neo expression plasmid alone served as control. Cells were fixed and then stained with DAPI (blue) at 48 h post transfection. Scale bar = 10 μm.

Next, we confirmed the interaction characteristics of pDNAJB6 with PCV2 Cap. Co-immunoprecipitation assays with either anti-GFP or anti-Flag antibodies showed that overexpressed exogenous Flag-pDNAJB6 did interact with recombinant GFP-Cap proteins (Figures 2A and B); Endogenous pDNAJB6 proteins were also detected in the immunoprecipitated complex in PCV2-infected cells or Cap-expressing cells, when the cell lysate was immunoprecipitated using the anti-Cap antibody (Figures 2C and D). GST pull-down assay showed that pDNAJB6 directly interacted with Cap (Figure 2E). These results demonstrated that both exogenous and endogenous pDNAJB6 interact with the Cap protein of PCV2. Furthermore, we examined whether the presence of PCV2 Cap could influence the localization of pDNAJB6 in cells. PK-15 cells were transfected with plasmids expressing Flag-pDNAJB6 and EGFP or plasmids expressing Flag-pDNAJB6 and GFP-Cap, and observed under laser-scanning confocal microscopy. Imaging analysis showed that Flag-pDNAJB6 appeared in both nucleus and cytoplasm in cells without GFP-Cap protein, but most of them were co-localized with GFP-Cap in the nucleus of cells expressed GFP-Cap, only part of them localized in the cytoplasm (Figure 2F).

### Knockout of pDNAJB6 inhibits the formation of autophagosomes and viral replication in PCV2-infected cells

**Figure 3:**
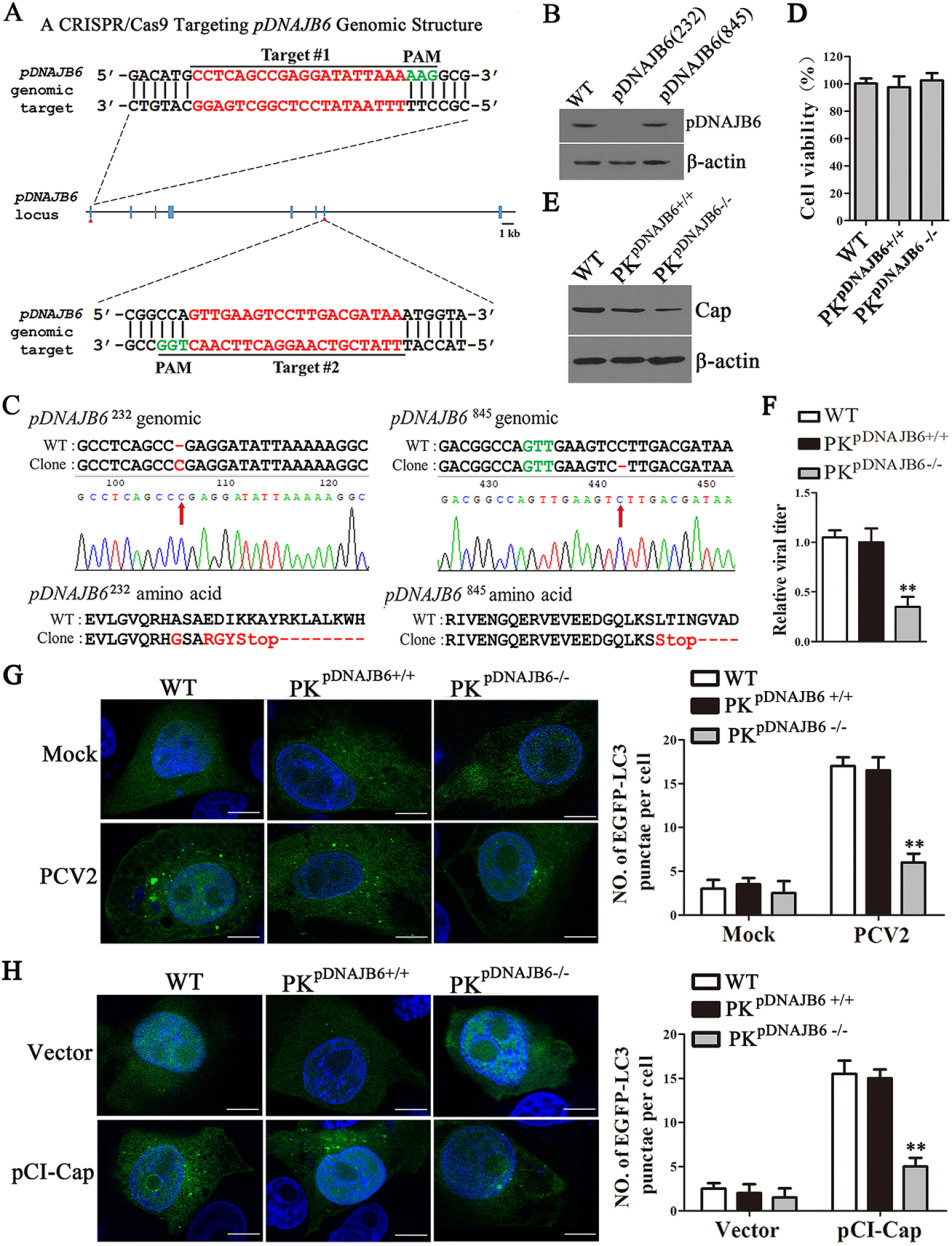
**Knockout of pDNAJB6 inhibits the formation of autophagosomes and viral replication in PCV2-infected cells. A** Schematic chromatogram representation of sgRNA targeting at the pDNAJB6 genomic region. PAM sequences are underlined and highlighted in green. sgRNA targeting sites are underlined and highlighted in red. Red arrows indicate gRNA targeting sites. **B** Western blot analysis of the pDNAJB6 expression in PK-15 cells infected with CRISPR/Cas9 lentivirus and then selected by puromycin. The lentiviruses contain gRNA-232 and gRNA-845. **C** Sequencing of pDNAJB6 locus amplified from the gRNA-232 (*pDNAJB6*^*2*32^) and gRNA-845 (*pDNAJB6*^845^) generated cell clone. Red arrows indicate insertions or mutations. Red dashes indicate deleted bases or amino acids. Red characters indicate inserted base or mutated amino acids. **D** Cell viability of cell lines stably knockout for pDNAJB6. **E, F** Knockout of pDNAJB6 inhibits PCV2 progeny virion production. Wild-type PK-15, 845PK^pDNAJB6+/+^ and 232PK^pDNAJB6−/−^ cells were infected with PCV2 (MOI = 5) for 48 h. PCV2 Cap were detected by Western blot (**E**), and relative fold-change in PCV2 titers were determined by TCID_50_ assay (**F**). **G, H** Knockout of pDNAJB6 inhibits the accumulation of autophagosomes induced by PCV2 infection or PCV2 Cap protein in PK-15 cells. PK-15, 845PK^pDNAJB6+/+^ and 232PK^pDNAJB6−/−^ cells stably expressing EGFP-LC3 were infected with PCV2 (MOI = 1) (**G**) or transfected with pCI-Cap (**H**) for 24 h. Scale bar = 10 μm. The right panel graph shows the number of autophagosomes by taking the average number of green puncta in 60 cells. Results are shown as the mean ± SD (*n* = 3). **p < 0.01 versus wild-type PK-15 cells with the same treatment.

To determine the roles of pDNAJB6 in the infectious process of PCV2, we constructed pDNAJB6 gene knockout PK-15 cells using CRISPR/Cas9 genomic editing system. Two guide RNAs (gRNA-232 and gRNA-845) were designed to target two sites of the exon 1 and exon 7 of pDNAJB6 genome sequences, respectively (Figure 3A). Single cell clones pDNAJB6 (232) and pDNAJB6 (845) were selected from cells infected with Cas9 recombinant lentivirus encoding gRNA-232 or gRNA-845, respectively. Western blot analysis showed that pDNAJB6 expression was deficient in the pDNAJB6 (232) cell clone, but was not affected in the pDNAJB6 (845) cell clone (Figure 3B). To further examine the genomic mutation of targeted locus, two pairs of primers flanking two gRNA target sites were designed to perform PCR amplification. Genomic sequencing of gRNA-232 cell clone (232PK^pDNAJB6−/−^) showed that a single nucleotide insertion leading to the alanine 15 site mutation to glycine, the glutamine 18 site mutation to arginine, the aspartic 19 site mutation to glycine, the isoleucine 20 site mutation to tryptophan, and resulting in an early stop codon (left). Genomic sequencing of gRNA-845 cell line (845PK^pDNAJB6+/+^) displayed a single nucleotide deletion leading to the leucine 227 site mutation to serine and resulting in an early stop codon (right) (Figure 3C). The cell viability assay showed that the viability of 232PK^pDNAJB6−/−^ and 845PK^pDNAJB6+/+^ cells were like wild-type PK-15 (WT) cells (Figure 3D). Next, wild-type PK-15, 232PK^pDNAJB6-/-^ and 845PK^pDNAJB6+/+^ cells were infected with equal amounts of PCV2 (MOI = 1) for 24 h or 48 h. PCV2 Cap expression and viral titers significantly decreased in 232PK^pDNAJB6-/-^ cells compared with wild-type PK-15 (WT) and 845PK^pDNAJB6+/+^ cells at 48 hpi (Figures 3E and F). Meanwhile, we noticed that the number of autophagosomes was lower in 232PK^pDNAJB6-/-^ cells than in wild-type PK-15 (WT) and 845PK^pDNAJB6+/+^ cells at 24 hpi (Figure 3G). Consistently, in the cells transfected with plasmids expressing PCV2 Cap, pDNAJB6 deficiency significantly reduced the formation of autophagosomes induced by Cap expression (Figure 3H). These results suggested that pDNAJB6 knockout inhibits the formation of autophagosomes and leads to decreased progeny virion production in PCV2-infected cells

### Overexpression of pDNAJB6 promotes the formation of autophagosomes and progeny virion production in PCV2-infected cells

**Figure 4:**
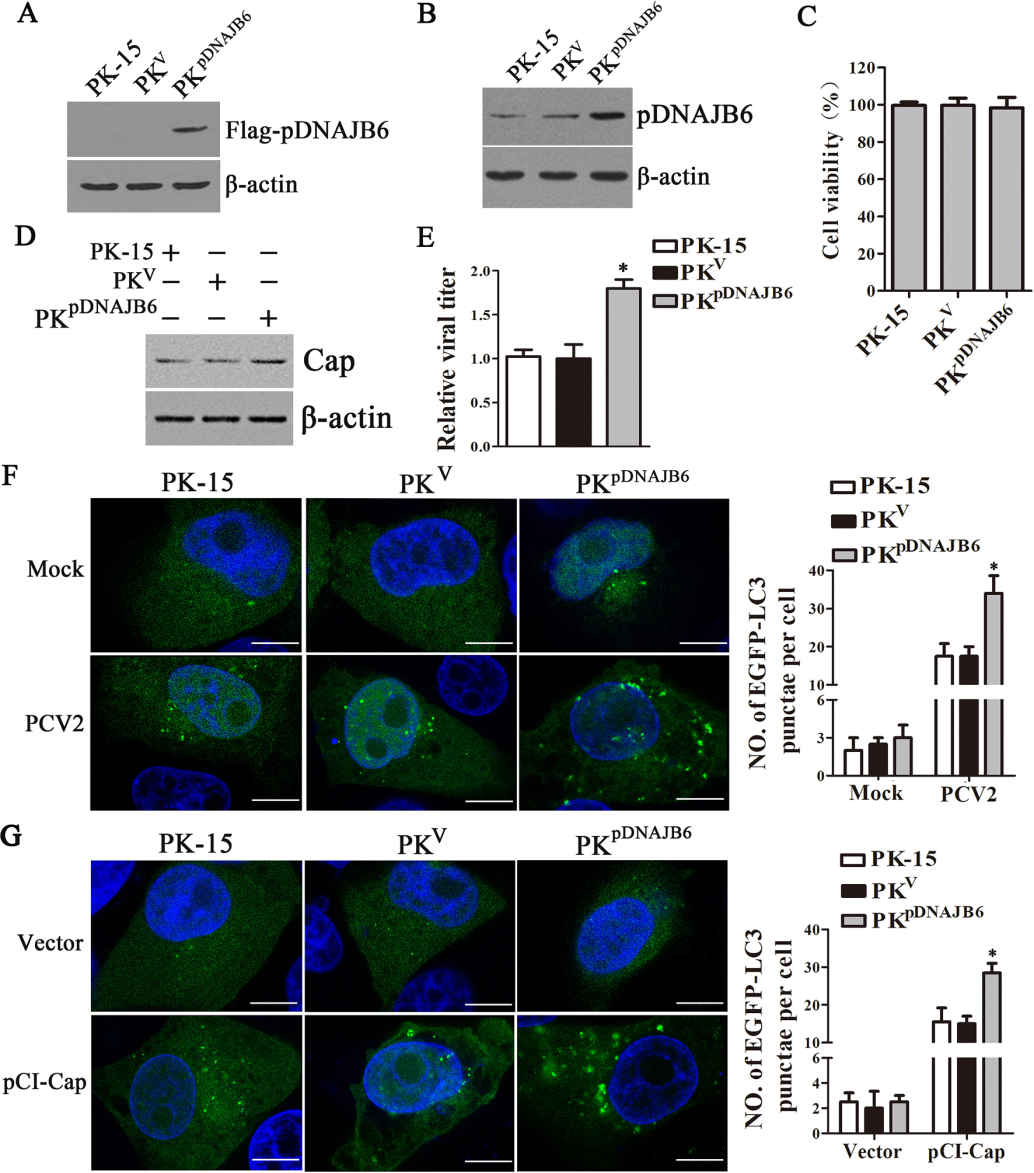
**Overexpression of pDNAJB6 promotes the formation of autophagosomes and progeny virion production in PCV2-infected cells. A, B** Western blot analyzes of pDNAJB6 protein levels in PK-15 cells with pDNAJB6 overexpression using anti-Flag antibodies (**A**) or anti-DNAJB6 antibodies (**B**). PK-15 are the cells without transfection, PK^V^ the cells transfected with blank vector, PK^pDNAJB6^ the cells transfected with the vector expressing Flag-pDNAJB6. **C** Cell viability of cell lines stably overexpressing pDNAJB6. **D, E** Overexpression of pDNAJB6 promotes PCV2 progeny virion production. Wild-type PK-15, PK^V^ and PK^pDNAJB6^ cells were infected with PCV2 (MOI = 5) for 48 h. PCV2 Cap were detected by Western blot (**D**). PCV2 titers were determined by TCID_50_ assay (**E**). **F, G** Overexpression of pDNAJB6 promotes the accumulation of autophagosomes induced by PCV2 Cap protein or PCV2 infection in PK-15 cells. PK-15 and PK^pDNAJB6^ cells that stably express EGFP-LC3 were infected with PCV2 (**F**) or transfected with pCI-Cap plasmid (**G**) for 24 h. Scale bar = 10 μm. The right panel graph shows the number of autophagosomes by taking the average number of green puncta in 60 cells. Results are shown as the mean ± SD (*n* = 3). *p < 0.05 versus wild-type PK-15 cells with the same treatment.

To further confirm the roles of pDNAJB6 in PCV2-induced autophagy and viral replication, we established a PK-15 cell line stably expressing Flag-pDNAJB6 (PK^pDNAJB6^) and a PK-15 cell line transfected with blank vector (PK^V^) as negative control. pDNAJB6 expression increased in PK^pDNAJB6^ cells compared to PK^V^ cells or wild-type PK-15 cells (Figures 4A and B). The cell viability assay showed that the viability of overexpressing pDNAJB6 cells was not significantly different from wild-type cells (Figure 4C). When these cells were infected with same dose of PCV2 for 48 h, Cap protein expression significantly increased in the cells overexpressing pDNAJB6 relative to the cells without pDNAJB6 overexpression (PK-15 and PK^V^) (Figure 4D). Similarly, progeny virion production also increased in the cells overexpressing pDNAJB6 compared with the cells without pDNAJB6 overexpression (PK-15 and PK^V^) at 48 hpi (Figure 4E). Furthermore, we examined the effect of overexpressing pDNAJB6 on the number of autophagosomes induced by PCV2 or Cap. The results showed that pDNAJB6 overexpression significantly increased the number of autophagosomes in either PCV2-infected cells or Cap expressing cells (Figures 4F and G). These results further indicate that pDNAJB6 plays an important role in enhancing the formation of autophagosomes and progeny virion production in PCV2-infected cells.

### The J domain of pDNAJB6 is essential for its interaction with Cap

**Figure 5:**
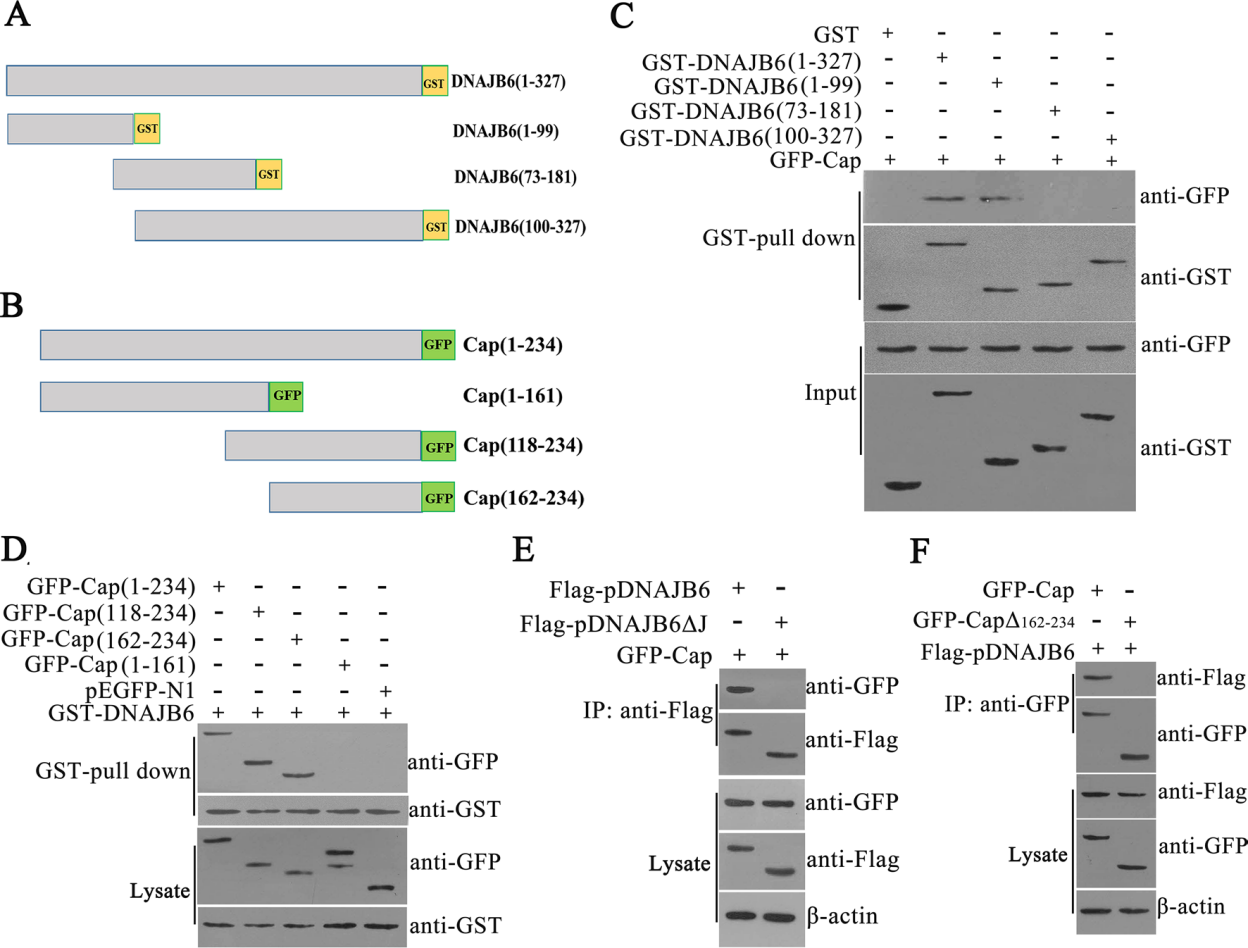
**The J domain of pDNAJB6 is essential for its interaction with Cap. A, B** Schematic representation of mutants of Cap and pDNAJB6. **C, D** GST pull-down assay. GST or GST fusion proteins were incubated with the indicated proteins and the bound proteins were analyzed by SDS-PAGE. GFP-tagged proteins and GST-tagged proteins were detected by Western blot. **E, F** The J domain of pDNAJB6 and the fragment (162-234 aa) of Cap are required for the interaction of pDNAJB6 with Cap. HEK-293T cells were transfected with the indicated plasmids, and cell lysates were immunoprecipitated with anti-Flag or anti-GFP antibodies followed by immunoblots using anti-GFP or anti-Flag antibodies.

To identify the regions of pDNAJB6 and Cap that were responsible for the specific interaction, a series of deletion mutants of pDNAJB6 and Cap were constructed (Figures 5A and B). GST pull-down assay showed that pDNAJB6 fragments 73-181 and 100-327 were not able to bind to Cap protein, whereas fragment 1-99 was able to interact with Cap protein, as well as the full length of pDNAJB6, suggesting that J domain (amino acid 1-99) of pDNAJB6 is required for interaction with Cap (Figure 5C). Meanwhile, the fragments 118-234 and 162-234 in the C-terminus of Cap interacted with pDNAJB6, while the other fragments of Cap failed to interact with pDNAJB6. This data suggested that the C-terminal (amino acid 162-234) of Cap is essential for the interaction of Cap with pDNAJB6 (Figure 5D). To further confirm the critical binding domain for the interaction of Cap with pDNAJB6, the expression vectors of Cap (162-234 aa) deletion mutant (CapΔ162-234) and pDNAJB6 J domain deletion mutant (pDNAJB6ΔJ) were constructed. Then, pDNAJB6ΔJ and Cap-GFP, or CapΔ162-234 and Flag-pDNAJB6 were co-expressed in PK-15 cells. IP assay showed that CapΔ162-234 deletion mutant lost the ability to interact with pDNAJB6, while pDNAJB6ΔJ deletion mutant lost the ability to interact with Cap (Figures 5E and F). Taken together, these data demonstrate J domain of DNAJB6 (1–99 aa) and the fragment (162-234 aa) of Cap are required for the interaction between pDNAJB6 and Cap.

### The interaction between pDNAJB6 and Cap promotes the production of autophagosomes induced by PCV2 infection

**Figure 6:**
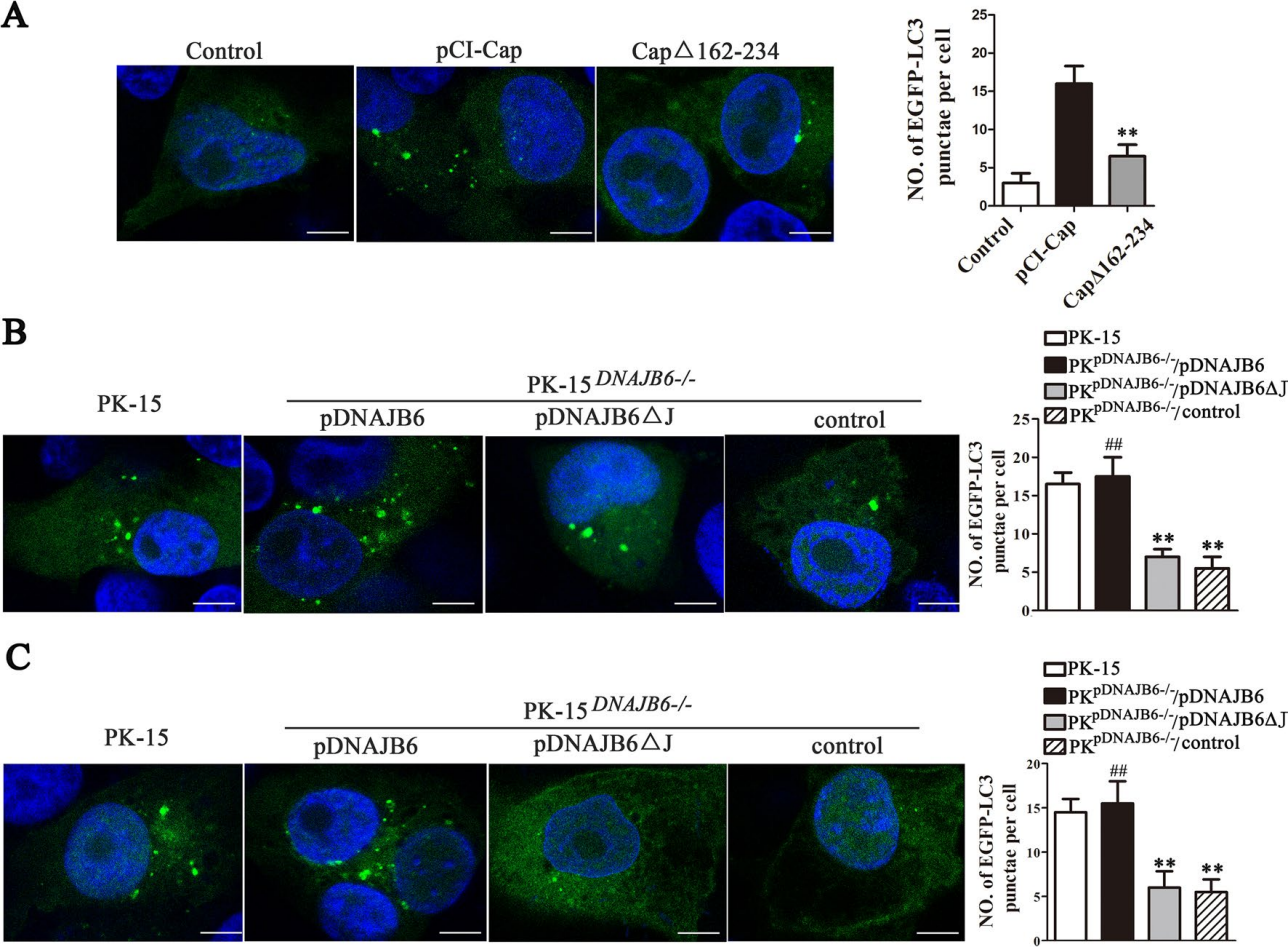
**The interaction between pDNAJB6 and Cap promotes the production of autophagosomes induced by PCV2 infection. A** Wild-type PK-15 cells stably expressing EGFP-LC3 were transfected with pCI-neo, pCI-Cap or pCI-CapΔ162-234 (CapΔ162-234) plasmid for 24 h, respectively. The average number of green puncta was counted and calculated in 60 cells for each group. Results are shown as the mean ± SD (*n* = 3). **p < 0.01 versus pCI-Cap vector transfected cells. **B, C** PK-15 and PK^pDNAJB6−/−^ cells stably expressing EGFP-LC3 were transfected with pCI-pDNAJB6, pCI-pDNAJB6ΔJ or blank vector (control) for 24 h, then cells were infected with PCV2 (**B**) or transfected with pCI-Cap plasmid (**C**) for 24 h. The average number of green puncta was counted and calculated in 60 cells in each group. Results are shown as the mean ± SD (*n* = 3). **p < 0.01 versus wild-type PK-15 cells. ^*##*^*p* < 0.01 versus PK^pDNAJB6−/−^ cells that transfected with blank vector (PK^pDNAJB6−/−^/control).

As pDNAJB6 overexpression could increase the number of autophagosomes in PCV2-infected cells or Cap-expressing cells, we supposed that pDNAJB6 might depend on the interaction with Cap to enhance the formation of autophagy in PCV2-infected cells. To verify this hypothesis, we observed and compared the number of autophagosomes in the cells respectively transfected with control plasmid (pCI-neo), full-length Cap expression plasmid (pCI-Cap), and CapΔ162-234 (mutated Cap that deleted pDNAJB6-binding domain) expression plasmid (pCI-CapΔ162-234). The results showed that puncta formation of GFP-LC3-labeled vesicles was significantly reduced in the cells expressing CapΔ162-234 compared to the cells expressing full-length Cap (Figure 6A). Similarly, in pDNAJB6 deficient (PK^pDNAJB6−/−^) cells, the numbers of autophagosomes markedly decreased relative to wild-type PK-15, when these cells were transfected with full-length Cap expression plasmid (pCI-Cap) or infected with PCV2 (Figures 6B and C); expression of full-length pDNAJB6 markedly increased the autophagosome numbers of PK^pDNAJB6−/−^ cells induced by PCV2 or Cap protein, and reached the level of wild-type PK-15 cells, yet expression of pDNAJB6 J domain deletion mutant (pDNAJB6ΔJ) was not able to increase the autophagosome numbers of PK^pDNAJB6−/−^ cells induced by PCV2 or Cap protein (Figures 6B and C). These results suggest that the interaction between pDNAJB6 and Cap can enhance the formation of autophagosomes during the process of PCV2 infection.

### pDNAJB6 interaction with Cap promotes PCV2 replication by autophagy increase

**Figure 7:**
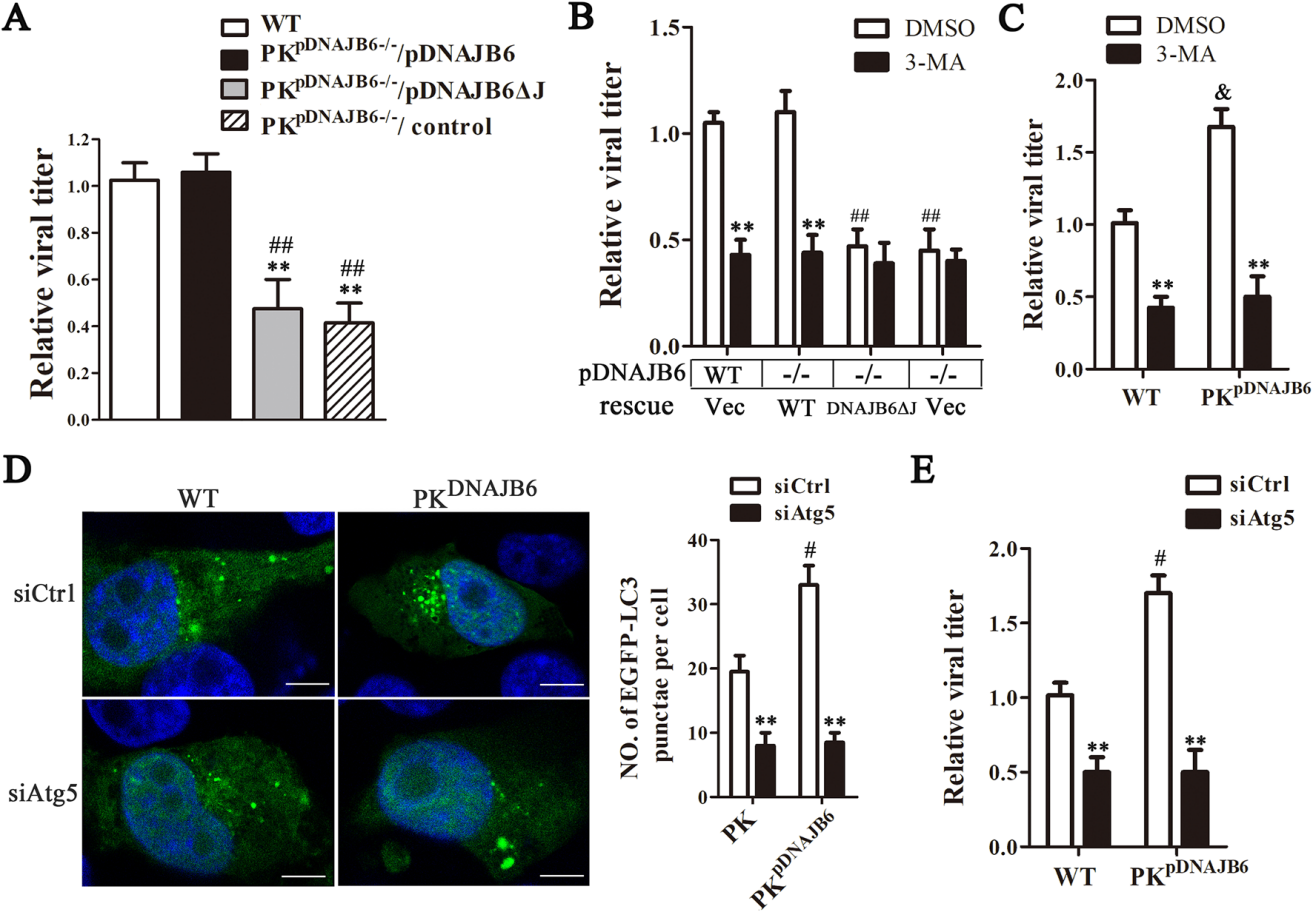
**pDNAJB6 interaction with Cap promotes PCV2 replication through enhancing of autophagy. A** Disruption of pDNAJB6/Cap interaction impedes PCV2 replication. PK^pDNAJB6−/−^ cells (transfected pCI-pDNAJB6 or pCI-pDNAJB6ΔJ for 12 h), wild-type PK-15, and PK^pDNAJB6−/−^ cells (transfected with pCI-neo (control)) were infected with PCV2 for 48 h, respectively. The relative production of progeny viruses was measured by TCID_50_ and calculated. **p < 0.01 versus wild-type PK-15 cells. ^##^*p* < 0.05 versus PK^pDNAJB6−/−^ cells transfected with full-length pDNAJB6 expression plasmid. **B, C** The effects of an autophagy inhibitor on the production of progeny virions. After pretreatment with or without 3-MA (5 mM), cells were respectively transfected with the indicated plasmids, and then the cells were infected with PCV2 for 48 h. Relative viral production was measured and calculated. **p < 0.01 versus DMSO treated cells that transfected with same plasmid; ^##^*p* < 0.01 versus PK^pDNAJB6−/−^ cells rescued wild-type pDNAJB6 with same treatment. ^&^*p* < 0.05 versus wild-type PK-15 cells with same treatment. **D, E** Effect of *atg5* silencing on the number of autophagosomes induced by PCV2 infection and progeny PCV2 production in cells infected with PCV2. The cells were transfected with control siRNA (siCtrl) and *atg5* siRNA (siAtg5), then infected with PCV2. After infection with PCV2 for 24 h, the average number of green puncta was counted and calculated in 60 cells in each group (**D**). After infection with PCV2 for 48 h, the relative production of progeny virions was measured and calculated **(E)**. Results are expressed as the mean ± SD (*n* = 3). **p < 0.01 versus the same type of cells transfected with siCtrl; ^#^*p* < 0.05 versus wild-type PK-15 cells with the same treatment.

Since the interaction of pDNAJB6 and Cap affects the number of autophagosomes induced by PCV2 or Cap, and autophagy promotes the replication of PCV2, we thus further determine the role of pDNAJB6/Cap interaction in PCV2 replication. Wild-type PK-15, PK^pDNAJB6−/−^ cells, and PK^pDNAJB6−/−^ cells expressing full-length pDNAJB6 or mutated pDNAJB6ΔJ were infected with equal amounts of PCV2 for 48 h. Comparison of progeny viral production showed that viral titers were significantly lower in either PK^pDNAJB6−/−^ cells or the PK^pDNAJB6−/−^ cells expressing pDNAJB6ΔJ than in wild-type PK-15 and the PK^pDNAJB6−/−^ cells expressing full-length pDNAJB6 at 48 hpi (Figure 7A). This suggested that abolishing pDNAJB6/Cap interaction impedes the replication of PCV2. Notably, in pDNAJB6 deficient (PK^pDNAJB6−/−^) cells, rescuing of full-length pDNAJB6 still was not able to increase the production of progeny virions when these cells were pretreated with autophagic inhibitor 3-Methyladenine (3-MA) (Figure 7B), indicating that enhancing effect of pDNAJB6 on the replication of PCV2 not only depends on pDNAJB6/Cap interaction, but also depends on the formation of autophagy. To further confirm this viewpoint, we detected the effects of inhibiting autophagy on the progeny virion production in PK^pDNAJB6^ cells that over-expressed pDNAJB6. Results showed that 3-MA markedly decreased viral production in both PK^pDNAJB6^ and PK-15 cells, and PK^pDNAJB6^ cells showed a similar capacity as PK-15 cells in viral replication in the presence of 3-MA (*P* > 0.05) (Figure 7C). However, we noted that the progeny viral production was markedly higher in PK^pDNAJB6^ cells than that in PK-15 cells when these cells were not treated with 3-MA (Figure 7C), as we observed in the above experiments. Consistent with these observations, silencing of Atg5 autophagy-related gene 5 (*atg5*) by transfection of atg5 specific siRNA (no significant effect on cell viability) inhibited the formation of autophagosomes, resulting in reduced progeny PCV2 production compared with control siRNA transfection in both PK^pDNAJB6^ and PK-15 cells (Figures 7D and E). Similarly, PK^pDNAJB6^ cells did not differ from PK-15 cells in progeny PCV2 production when these two cells were transfected with same concentration of *atg5* specific siRNA then followed by same doses of PCV2 infection (Figure 7E). These results suggest that the interaction of pDNAJB6 with Cap promotes PCV2 replication through enhancement of autophagy formation.

## Discussion

Once viruses invade host cells, the host cells initiate a range of antiviral mechanisms to prevent the virus from infection, such as innate immunity, autophagy and ubiquitin–proteasome system [5, 27, 33, 34]. However, the virus can employ host factors to promote viral infection, during which some cellular proteins play important roles. During PCV2 infection, cellular proteins including gC1qR, NPM1, MKRN1, Hsp40, Par4 and Nap1 have been identified to be involved in the regulation of viral replication in our and other previous studies [5, 12, 32]. DNAJB6, a member of HSP40 family, is an abundant cytosol/nucleus protein consisting in three domains: J domain, Gly/Phe-rich domain, and a C-terminal region, in which the J domain of approximately 70 amino acids in size is highly conserved among different species. Porcine DNAJB6 (pDNAJB6) is encoded by the porcine *DNAJB6* gene and possesses 84.58% identity to human DNAJB6 (GenBank: KJ892903.1). In this study, pDNAJB6 could be detected in most tissues of pigs, such as heart, liver, spleen, lung, kidney, and lymph nodes (Data not shown), and expression of porcine DNAJB6 (pDNAJB6) was efficiently upregulated with PCV2 loads. Importantly, we determined the roles of pDNAJB6 as a PCV2 Cap-binding protein that promotes PCV2 replication via regulation of the autophagy of host cells.

Autophagy plays different roles in many different diseases and processes [35]. Autophagy plays an antiviral role in the infectious process of some viruses, such as foot-and-mouth disease virus and sindbis virus [27, 36]. Otherwise, autophagy is used by viruses to help their own replication, as for the porcine reproductive and respiratory syndrome virus [30, 31]. In previous studies, PCV2 has been identified to trigger autophagosome formation and enhance autophagic flux to promote virus replication [31]. Therefore, we tested the effects of pDNAJB6 knockout or overexpression on the formation of autophagy caused by PCV2 or PCV2 Cap in PK-15 cells. Interestingly, we found that knockout of pDNAJB6 inhibited the formation of autophagosomes induced by PCV2 infection or PCV2 Cap protein. By contrast, overexpression of pDNAJB6 resulted in the opposite effects. We confirmed that pDNAJB6 promotes the formation of PCV2/Cap-induced autophagosomes.

Hsp40 family members play important roles in the life cycles of multiple range of viruses. In many cases, Hdj2, hTid-1, Hdj1 and Hsp40B1 promote the replication of JEV, HSV-1, HIV-2 and influenza A virus, respectively [17, 18, 20, 21]. However, Hsp40A1, B1, B6, and C5 inhibit the replication of HIV-1 [37], and Hdj2 and Hdj1 also inhibit the replication of HBV [13, 15]. To identify the effects of pDNAJB6 responsible for PCV2 replication, we found that knockout of pDNAJB6 reduces progeny virus production, but overexpression pDNAJB6 enhances PCV2 replication. Thus, we confirmed that pDNAJB6 promotes PCV2 replication.

PCV2 promotes self-replication by increasing the formation of autophagosomes. However, it is unclear whether it depends on the interaction between Cap and pDNAJB6 in this process. To further identify the region of pDNAJB6 responsible for these processes, we determined the binding regions of Cap and pDNAJB6 by GST Pull-Down assays. As shown, J domain of DNAJB6 (1–99 aa) and the fragment (162-234 aa) of Cap are required for the interaction between pDNAJB6 and Cap, we obtained mutation vectors for deleting the interaction region of DNAJB6 and Cap, respectively. The effects of DNAJB6 mutation on PCV2-induced autophagosomes and PCV2 titers were investigated by transfecting mutant or wild type DNAJB6 into DNAJB6 knockout PK-15 cells. The results demonstrate that the formation of autophagosomes depends on the interaction between pDNAJB6 and Cap.

Next, we further determined the role of pDNAJB6/Cap interaction in PCV2 replication. We added the full-length and mutant interaction region plasmids to the pDNAJB6-deficient cells to compare the viral titers in the cells at the same PCV2 infection time points, and found that abolishing pDNAJB6/Cap interaction impeded the replication of PCV2. To verify that the DNAJB6-Cap interaction promotes PCV2 replication by autophagy, we treated the cells with the autophagy inhibitor 3-MA. The results indicated that the enhancing effect of pDNAJB6 on the replication of PCV2 not only depended on pDNAJB6/Cap interaction, but also depended on the formation of autophagy. As has been known, 3-MA is a widely used inhibitor of autophagy via its inhibitory effect on class III PI3K at early autophagic processes, and *atg5* gene silencing can also inhibit autophagy in the early stages [30, 38, 39]. When we silenced the *atg5* gene in both wild type and overexpressing pDNAJB6 gene cells, the number of autophagosomes and the PCV2 titers significantly decreased. These results suggest that the interaction of pDNAJB6 with Cap promotes PCV2 replication through enhancing autophagosome formation.

In summary, the data presented here demonstrate that DNAJB6 promotes PCV2 replication through interaction with Cap proteins to increase the number of autophagosomes. Meanwhile, the interaction of DNAJB6 and Cap is indispensable for promoting the formation of autophagosomes caused by PCV2 Cap and further enhances the autophagy flow in cells. This study presented a novel role of DNAJB6 in regulation of porcine circovirus replication, which might provide a molecular basis for anti-PCV2 treatments.
